# The Relationship between Intelligent Image Simulation and Recognition Technology and the Health Literacy and Quality of Life of the Elderly

**DOI:** 10.1155/2022/9984873

**Published:** 2022-02-23

**Authors:** Baojian Wei, Chunyu Li, Jiangye Xu

**Affiliations:** ^1^Yanbian University, Yanji, China; ^2^Shandong First Medical University and Shandong Academy of Medical Sciences, Taian, China; ^3^School of Mechanical Electrical and Information Engineering, China University of Mining and Technology Beijing Campus, Beijing 100083, China

## Abstract

In order to explore the relationship between intelligent image recognition technology and the mentality and quality of life of the elderly, this paper combines intelligent image simulation technology to identify the behavior of the elderly, protect the safety of the elderly, and provide timely feedback on the adverse conditions of the elderly. Moreover, this paper improves the traditional intelligent image recognition algorithm, verifies the research method of this paper through experimental research, and puts forward corresponding suggestions. Through investigation and research, we can see that the level of health literacy of elderly patients with chronic diseases is low. Therefore, in the future health education, we should strengthen health education for elderly patients with chronic diseases, use different mass media to propagate health knowledge, and promote the formation of healthy lifestyles and behaviors for elderly patients with chronic diseases. At the same time, the experiment also verified that the intelligent image recognition technology proposed in this paper has a positive effect in improving the mentality and quality of life of the elderly.

## 1. Introduction

With the rapid economic development, all walks of life in our country have taken on a new look; people's living standards have also been rapidly improved; and the overall development has shown a prosperous situation. However, we also have to admit that in the process of development, there are still many problems that need to be summarized and resolved, such as environmental protection issues, population structure issues, urban-rural gap issues, development balance issues, industrial structure adjustment issues, and so on. Among them, the problem of population structure is particularly prominent, and as an important part of our country's population, the problems of the elderly are worthy of in-depth study. In particular, with the increasing aging of our country, the resulting problems and actual impacts have become more and more obvious so that social development has been restricted on many occasions. Therefore, it is urgent to solve the aging problem [1].

In today's society, a common phenomenon is that families are miniaturized, there are more and more only children, and the burden of caring for disabled elderly people is getting heavier. Therefore, children urgently need new technologies and new things that can help them take care of the elderly, and disabled elderly people urgently need new things and new technologies that can assist them in their daily lives. If the control of external devices can be combined with the EEG signals of the elderly, it will not only regain their confidence in life and reduce the burden on children but also make meaningful explorations and attempts for the application and promotion of EEG research and development in real life [2]. Health literacy is the ability of individuals to acquire, process, and understand basic health care knowledge and services needed to make appropriate health decisions. Moreover, as an important part of health education and health promotion, health literacy has gradually attracted attention from domestic and foreign scholars in recent years [3].

The framework of this article is as follows:

With the rapid economic development, all levels of Chinese society have new faces; people's living standards are rising rapidly; and the overall development is developing in a prosperous environment. But we must also admit that there are many problems in the development process, which need to be summarized and solved, such as environmental problems, demographic structure, differences between cities and network problems of balanced development, industrial structure changes, and other especially important parts of the population problem of the elderly population, worthy of in-depth study. In particular, as our country ages, problems and practical consequences become increasingly apparent, and social development is in many cases hindered. Therefore, the solution to the aging problem is inevitable (1).

A common phenomenon in today's society is the miniaturization of families, the increasing number of children, and the increasing burden of caring for the elderly. Therefore, children desperately need new technologies and things to help them care for the elderly, while disabled people need new and new technologies to help them get through their daily lives. If combined with the control of external devices and electronic brains of the elderly, these indicators not only rebuild their lives and reduce the burden on the children's confidence but also can be related to the research and development of electronic brain attempts in real life quality (2). Health is a person's ability to access, treat, and understand the basic knowledge and services required for appropriate decision-making in health care. In addition, as an important part of health education and health promotion, health self-improvement has gradually attracted the attention of scientists at home and abroad in recent years.

## 2. Related Work

In the past research work, people mostly used traditional sensing methods such as wearable devices and cameras to recognize indoor movements. Among them, the more typical example is the use of accelerometers, gyroscopes, or their fusion system to recognize action behavior [4]. The advantage of this type of wearable device is that it is highly robust to changes in the external environment. Secondly, researchers also use various types of cameras, such as depth cameras, to track and reconstruct human nonrigid motion [5]. This method can capture a large amount of external information, and the upper limit of performance is very high. The investigation in literature [6] shows the rejection of invasive devices by the elderly. Moreover, the camera has high requirements for lighting conditions, is easy to leak privacy, and is not suitable for use in an environment with high privacy requirements such as home. The main principle of using radar for action recognition is the micro-Doppler feature [7]. The action of the target will change the characteristics of the radar echo, and the activities of various parts of the body are different when different actions occur. If the human body is regarded as a collection of a series of reflection points, the overall frequency distribution finally reflected by these reflection points will correspondingly differ around the main modulation band. Researchers used to extract the required micro-Doppler features mainly based on the time-Doppler map (TDM). The most common are artificially predefined physical characteristics, including changes in the time-frequency spectrum envelope caused by the physical characteristics of the action [8]. This physical feature is easily affected by changes in the external environment (such as human height, weight, etc.) and often requires experts in related fields to design, which is time-consuming and labor-intensive. In order to solve these problems, a series of feature definition methods in other similar fields are transplanted into this task, including classic wavelet-based features [9] and Mel-frequency cepstrum coefficient (MFCC) [10] commonly used in speech analysis. The existence of these features simplifies the step of manually defining features but relatively also makes a certain compromise in classification performance. Since the different representations of radar data [11] can also be regarded as a category of generalized images, advanced deep learning techniques in the image field can be used to automatically extract low-dimensional abstract features from it. Literature [12] used an unsupervised pretrained sparse autoencoder to extract features from TDM and achieved an 87% recognition rate in fall detection. Literature [13] used a convolutional autoencoder to replace the traditional sparse autoencoder, borrowed the idea of inception to design the network structure, and obtained a classification accuracy of 94.2% on 12 types of actions. In addition, there is also a more comprehensive use of optimized networks for human-action-recognition-related work [14]. However, one of the problems in these studies is that the signal representation domains used exist in a two-dimensional form, which cannot simultaneously characterize the time, space, distance, and Doppler frequency information contained in the radar signal, and it is bound to lose part of the information during action recognition.

In order to accomplish the goal of behavior inference, the most direct method is to install multiple sensors in the home environment. At present, the existing representative work includes the AAL behavior inference system that uses the depth camera to recognize actions and analyze context information [15] and the scheme of synchronizing the depth camera and wearable inertial sensor data proposed in literature [16]. Literature [17] used a variety of sensors (passive infrared sensors, door frame magnetic strips, and flow sensors) placed in various locations in the house to perceive the behavior of the monitored person in the entire indoor environment. This solution combines the prior knowledge of the sensor installation position to determine the behavior. For example, the doorframe magnetic stripe data can be used to infer the target's going out behavior, and the bathroom flow sensor data can be used to infer the target's bathing behavior. At the same time, some work focuses on the use of data mining methods to analyze mixed time series from multiple sensors and establish a person's daily behavior characteristics through the time changes of several quantitative or qualitative parameters [18]. Although such programs have achieved good results, their respective shortcomings are also obvious. For example, information-rich cameras and wearable sensors can cause privacy and intrusive issues when used for behavior inference and motion recognition. Moreover, the multisensor fusion scheme is very cumbersome to install in practical applications and requires specific protocols to coordinate with each other. This has led to this type of research can only be limited to the laboratory environment and far from being applied and promoted in the actual family [19].

## 3. Intelligent Image Simulation and Recognition Algorithm Applied in Medical Field

In practical applications, two convolution templates in horizontal and vertical directions are mostly used. Common ones are Sobel, Prewitt, and Scharr's horizontal convolution template *G*_*H*_(*x*, *y*) and vertical convolution template *G*_*V*_(*x*, *y*). Prewitt operator can suppress the influence of noise to a certain extent, but its detection ability is not strong. The Sobel operator considers that the importance of neighboring pixels to the current pixel is related to the spatial distance of the current pixel. Generally speaking, the closer the distance, the stronger the impact. The Scharr operator also assigns different importance to adjacent pixels according to the spatial distance relationship with the current pixel.

For a given behavioral image *I*(*x*, *y*), its gradient magnitude can be expressed as follows:

(1) $$\begin{array}{c} M\left(x,y\right)=\sqrt{M_{H}^{2}\left(x,y\right)+M_{V}^{2}\left(x,y\right)} \end{array}$$

where *M*(*x*, *y*) represents the gradient magnitude of the behavior image *I*(*x*, *y*) at the position (*x*, *y*). The direction of the gradient is the direction where the function *F* (*x*, *y*) changes the fastest. When there is an edge in the image, there must be a large gradient value. On the contrary, when there is a smooth part in the image, the gray value changes little, so the corresponding gradient is also small.

We can get the gradient image by computing the gradient of each pixel in the image. *M*_*H*_(*x*, *y*) and *M*_*V*_(*x*, *y*) are the horizontal and vertical gradient amplitude components of the behavior image at the point (*x*, *y*), respectively. The calculation method is as follows [20]:

(2) $$\begin{array}{c} M_{H}\left(x,y\right)=I\left(x,y\right)⊗G_{H}\left(x,y\right) \end{array}$$

(3) $$\begin{array}{c} M_{V}\left(x,y\right)=I\left(x,y\right)⊗G_{V}\left(x,y\right) \end{array}$$

The direction where the greatest change occurs in the gradient intensity field is called the gradient direction. From the horizontal and vertical gradient amplitude components, the gradient direction of this point can be defined as follows [21]:

(4) $$\begin{array}{c} D\left(x,y\right)=\arctan\frac{M_{V}\left(x,y\right)}{M_{H}\left(x,y\right)} \end{array}$$

If the *D*(*x*,*y*) of a pixel is different from its neighborhood, it means that the gradient direction of the pixel has changed.

The gradient magnitude of the behavior image can accurately extract the structural features of the behavior image, and the structure information of the behavior image is the human visual.

The principle of Shader is to use the threshold and UV to calculate the degree of transparency and then use LERP control to change the range of transparency, avoiding conditional judgment. Instead of creating a material for each image, you can use a C# script to dynamically create a material for Shader, which is also easier to use, more user-friendly, and easier to animate.

Why does the gradient information, which has been repeatedly successful in the field of natural behavior image quality evaluation, fail in the face of screen behavior image quality evaluation? This is a question worthy of our consideration. Different from natural behavior images, screen behavior images have the following characteristics:

1. The noncontinuous tone content in the screen behavior image is directly generated by the computer without the need for a collection process. Therefore, the area is completely noise-free.
2. Screen behavior images usually contain various forms of content, such as text, graphics, and natural behavior images.
3. The text area in the screen behavior image usually has many sharp edges and a large number of smooth areas, such as PowerPoint, e-magazines, and web pages.

**Figure 1:**
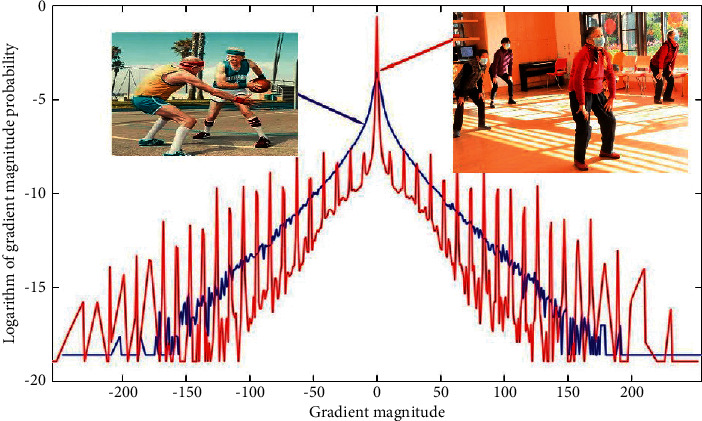
Comparison of the statistical characteristics of the gradient amplitude between the screen behavior image and the natural behavior image.

As shown in Figure 1, we counted and compared the statistical characteristics of the gradient amplitude of the screen behavior image and the natural behavior image. It can be observed from the figure that the gradient amplitude probability of the natural behavior image has a high degree of Kurtosis in the logarithmic domain, that is to say, it has a sharp peak at the zero point and a heavy-tailed distribution on both sides of the zero point (heavy-tailed distribution). The statistical characteristics of the gradient amplitude of the natural behavior image can be modeled using a generalized Laplace distribution. In comparison, the gradient amplitude of the screen behavior image exhibits severe oscillations in the “tail” part of the heavy tail distribution.

Although the existing gradient-based behavioral image quality evaluation models have general performance in evaluating screen behavioral image quality, these algorithms have a common defect: they only consider the gradient magnitude information and ignore the effect of gradient direction on behavioral image quality. The importance of evaluation: the gradient magnitude is a scalar, which can only represent the brightness change of a local area of the behavior image, and the gradient direction carries rich spatial information even in a smooth area where the brightness changes slowly.

**Figure 2:**
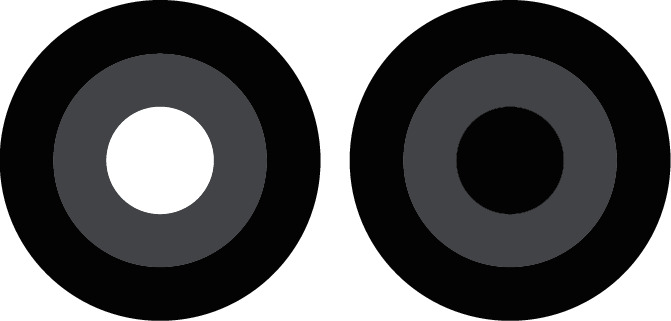
Gradient direction comparison.

The gradient direction information, in the form of gradient histogram, effectively solves the problem of object detection in the field of computer vision and digital behavior image processing. However, the gradient direction is important for the evaluation of behavioral image quality. In particular, the value of screen behavior image quality assessment has not been deeply explored. The traditional gradient direction calculation method is shown in formula (4), but this calculation method is unstable. To this end, we design a new gradient direction calculation method based on the local information of the gradient amplitude of the behavioral image. This method can better describe the change of gradient direction characteristics caused by behavioral image distortion (see Figure 2).

**Figure 3:**
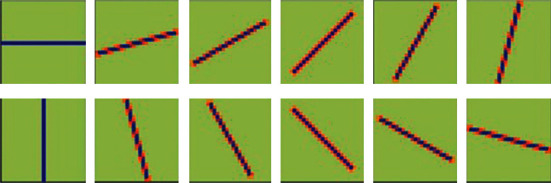
Convolution kernel in 12 directions.

As shown in Figure 3, we have considered a total of 12 angles from О to *x* at equal intervals, that is, *i* × *π*/12, where *i* ∈ {0,…, 11}. Therefore, for a specific screen behavior image, we can obtain 12 gradient patterns *D* in different directions. The gradient pattern of each fixed direction can be obtained by convolution operation between the convolution kernel *L*_1_ in the *i*-th direction and the gradient amplitude *M*(*x*, *y*) of the screen behavior image [22].

### 3.1. Test Method

So what is recognition? The so-called image recognition, as the name implies, is to make a variety of image processing, analyze, and finally identify the target we want to study. As we see in many pictures in lif, we humans can recognize planes, people, cars, traffic signs, and so on, and we can do much more with the information we collect.

“Computer image recognition” and “human image recognition” in principle and there is no essential difference, but “human image recognition” information processing is more complex, the computer lack of human perception and visual impact. In fact, we recognize objects not only by the memory of the whole image, but by recognizing the features of the image itself. The first is the image, which is recognized by every kind of characteristic image, but a lot of times we don't realize this. For example, when we see a rose, how do we know it is a rose? When we see a flower, we can tell from its features whether it has thorns or not, based on what we learned as children from our parents, teachers, books, and so on. Is it shaped like the petals of a rose? What is the color? What is the shape of the leaves? It goes on to identify whether the flower is a rose or some other kind of flower.

Up to now, there are many different technology methods of image recognition, which can be roughly divided into traditional image recognition method and fusion neural network algorithm recognition based on traditional image recognition. Neural network image recognition technology is a relatively new image recognition technology; the neural network here refers to the artificial neural network, that is to say, this neural network is not the real neural network of the animal itself but the artificial creation of a human imitation of animal neural network. In neural network image recognition technology, a deep learning model based on a convolutional neural network is a new star in the field of artificial intelligence and has made remarkable progress in many fields of artificial intelligence, especially in the field of image recognition.

The specific calculation steps are as follows:

For the specific screen behavior image *I*(*x*, *y*), the gradient pattern of the *i*-th direction is calculated as follows:

(5) $$\begin{array}{c} D_{1}\left(x,y\right)=L_{1}⊗M\left(x,y\right) \end{array}$$

where *M*(*x*, *y*) represents the gradient magnitude of the screen behavior image *I*(*x*, *y*) at the position (*x*, *y*) and can be expressed as follows:

(6) $$\begin{array}{c} M\left(x,y\right)=\left|M_{H}\left(x,y\right)\right|+\left|M_{V}\left(x,y\right)\right| \end{array}$$

where the symbol *||* represents the absolute value operation, and *M*_*H*_(*x*, *y*) and *M*_*V*_(*x*, *y*) represent the directional derivatives of the screen behavior image *I*(*x*, *y*) in the horizontal and vertical directions, respectively, and can be expressed as follows:

(7) $$\begin{array}{c} M_{H}\left(x,y\right)=I\left(x+1,y\right)-I\left(x,y\right) \end{array}$$

(8) $$\begin{array}{c} M_{G}\left(x,y\right)=I\left(x,y+1\right)-I\left(x,y\right) \end{array}$$

As shown in Figure 3, the convolution kernel *L* in each fixed direction can be obtained by rotating *L*_0_ by *i* × *π*/12. Through the calculation method shown in formula (5), After a set of 12 convolution kernels are convolved with the gradient amplitude *M*(*x*, *y*) of the screen behavior image, a corresponding set of 12 different directions of gradient pattern *D*_*i*_ is obtained. Compared with the method shown in formula (4), the proposed method takes into account a larger range of neighboring pixel values, so it has better stability, especially in the smooth area without sharp fluctuations.

After obtaining the gradient direction map of the 12 different directions of the screen behavior image *I*(*x*, *y*), the final gradient direction is by selecting the direction with the strongest response among the 12 directions of each pixel as the final gradient direction of the point [23]:

(9) $$\begin{array}{c} D\left(x,y\right)=n\times \frac{\pi }{12} \end{array}$$

(10) $$\begin{array}{c} n=\arg_{i}^{\max}\left\{D_{i}\left(x,y\right)\right\},i\in \left(0,\ldots ,11\right) \end{array}$$

The gradient directions of the reference and distorted screen behavior images are extracted, and the gradient direction similarity is defined as follows:

(11) $$\begin{array}{c} DS\left(x,y\right)=\frac{2D_{r}\left(x,y\right)D_{d}\left(x,y\right)+C_{d}}{D_{r}^{2}\left(x,y\right)D_{d}^{2}\left(x,y\right)C_{d}} \end{array}$$

where *D*_r_(*x*, *y*) and *D*_d_(*x*, *y*) represent the gradient pattern of the reference and distorted screen behavior images, respectively, and *C* is a positive number to prevent instability when the denominator is 0. In this experiment, *C*_*d*_=205 is used.

In order to generate a single quality index of the distorted screen behavior image, the partial quality map DS(*x*, *y*) needs to be merged. Since different areas have different effects on the overall perception of behavioral image quality, we use the standard deviation of DS(*x*, *y*) as the quality indicator of the distorted screen behavioral image:

(12) $$\begin{array}{c} DS\;S={\left(\frac{1}{N}\sum_{\left(x,y\right)\in \Omega }{\left(DS\left(x,y\right)-m_{DS}\right)}^{2}\right)}^{1/2} \end{array}$$

where Ω represents the collection of all pixels in the entire screen behavior image, *N* is the total number of pixels in the collection, and *m*_*DS*_ is the average value of DS(*x*, *y*). It should be pointed out that the larger the value of DSS, the deeper the distortion of the behavior image, which indicates that the perceived quality of the behavior image is worse.

Considering that the gradient magnitude can effectively describe the structural information of the behavior image, we use the gradient magnitude as an auxiliary feature and jointly consider the gradient direction and the gradient magnitude to describe the perceptual quality of the screen behavior image. Similarly, the similarity of gradient magnitude is defined as follows [24]:

(13) $$\begin{array}{c} MS\left(x,y\right)=\frac{2M_{r}\left(x,y\right)M_{d}\left(x,y\right)+C_{M}}{M_{r}^{2}\left(x,y\right)+M_{d}^{2}\left(x,y\right)C_{M}} \end{array}$$

where *M*_*r*_(*x*, *y*) and *M*_*d*_(*x*, *y*) are the gradient amplitude maps of the reference and distorted screen behavior images, respectively, and *C*_*M*_ is also a positive number to ensure the stability of the score. In this experiment, *C*_*M*_=160.

As mentioned earlier, DS(*x*, *y*) and MS(*x*, *y*) describe the similarity between reference and distorted screen behavior images from different aspects. In order to fully describe the similarity between the reference and distorted screen behavior images, we combine DS(*x*, *y*) and MS(*x*, *y*) together as follows:

(14) $$\begin{array}{c} GS\left(x,y\right)=\left[DS\;{\left(x,y\right)}^{\alpha }\right]\left[MS{\left(x,y\right)}^{\beta }\right]. \end{array}$$

where *α* and *β* are both positive numbers, which are used to adjust the relative importance of the similarity of the gradient direction and the similarity of the gradient amplitude in the proposed method. In particular, when *α*=0, only the gradient magnitude is used for quality evaluation. When *β*=0, only the gradient direction should be used for quality assessment. The gradient direction and the gradient magnitude have the same importance for the screen behavior image quality evaluation, so we set *α*=*β*=1 in this algorithm.

Finally, the standard deviation of the joint similarity is used as the behavioral image quality indicator:

(15) $$\begin{array}{c} GSS={\left(\frac{1}{N}\sum_{\left(x,y\right)\in \Omega }{\left(GS\left(x,y\right)-M_{GS}\right)}^{2}\right)}^{1/2} \end{array}$$

where *m*_*GS*_ is the average value of GS(*x*, *y*). According to the definition of GSS, the smaller the value of GSS, the greater the similarity between the reference and the distorted screen behavior image, and the better the quality of the distorted behavior image.

## 4. The Relationship between Intelligent Image Simulation and Recognition Technology and the Mentality and Quality of Life of the Elderly

Similar to biological systems, the main purpose of computer vision is to obtain machine behavior through possible “techniques” and “processing methods.”

Only the combination of complex “information processing tasks” and the “reasonable control and coordination” of these tasks can achieve image understanding.

The main purpose of computer vision is to use technically feasible “techniques” and “processing methods” to achieve machine behavior similar to the behavior of biological systems.

**Figure 4:**
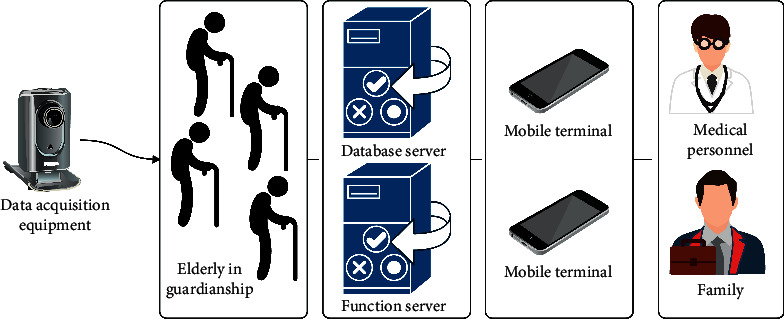
System architecture diagram.

Figure 4 shows the main architecture of the active nursing system for the elderly in this paper. The architecture adopts the C/S mode. The server provides services such as data storage and system interface. The client is used to view and monitor the activity information of the elderly under guard.

In the previous section, the system model of this paper has been constructed and applied to the real-time monitoring of the elderly, and the improvement of the quality of life of the elderly after the application of the model has been calculated. The effectiveness of the method in this paper is verified through experiments combined with questionnaire surveys.

The average score of health literacy of elderly patients with chronic diseases is (57.60 ± 21.96), and the scores of each dimension are shown in Table 1.

Depression is significantly negatively correlated with basic activities of daily living and functional activities of daily living and is significantly negatively correlated with health literacy. Basic and functional daily living abilities are significantly positively correlated with health literacy, as shown in Table 2.

The first step is the regression analysis of depression on daily activity ability to obtain the path coefficient *c*. The second step is the regression analysis of depression on health literacy to obtain the path coefficient *a*. In the third step, health literacy is included, and the regression analysis of depression and health literacy on daily activity ability is performed to obtain path coefficients *b* and *c*' (Table 3). The results found that depression and health literacy can significantly predict the ability of daily living. After adding health literacy, the *R*^2^ adjusted by the model increased from 0.324 to 0.521. The above results show that health literacy satisfies the conditions of the intermediary variables.

**Figure 5:**
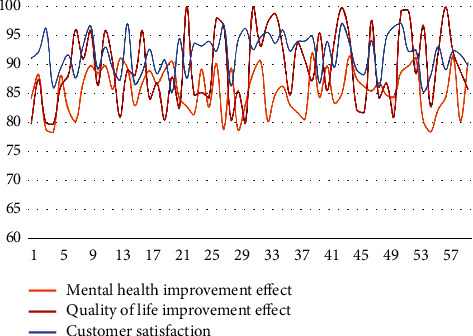
Statistical diagram of model effect evaluation.

Finally, the model of this paper is evaluated through expert evaluation, which mainly evaluates the effect of improving the mental health of the elderly, the effect of improving the quality of life, and customer satisfaction. The results are shown in Table 4 and Figure 5.

It can be seen from the above experimental research that the method proposed in this paper can effectively improve the mental health of the elderly and improve the quality of life of the elderly.

## 5. Conclusion

Due to the imperfect elderly care system in my country, the trend of aging, empty nesting, and chronic disease among the elderly has intensified. Moreover, symptoms such as hemiplegia and cerebral thrombosis are common in the elderly, which makes the long-term care of the elderly population more and more serious. In particular, the care of disabled elderly people needs to be solved urgently. Currently, elderly patients with chronic diseases have low levels of health literacy. Therefore, in the future health education, we should strengthen health education for elderly patients with chronic diseases, use different mass media to propagate health knowledge, promote the formation of healthy lifestyles and behaviors of elderly patients with chronic diseases, and fundamentally improve the health literacy level of elderly patients with chronic diseases. In addition, we can also use intelligent simulation recognition technology to recognize the behavior of the elderly and improve the monitoring effect of the elderly, so as to quickly respond to emergencies in a timely manner. Through experimental research, it can be known that the method proposed in this paper has good results.

## Figures and Tables

**Table 1:** Health literacy scores of elderly patients with chronic diseases (*n* = 382).

Dimension	Reference range	Score		$\bar{x}\pm s$
		Minimum	Max	
Total health literacy	0–96	28	89	57.60 ± 21.96
Information access capacity	0–36	14	32	21.64 ± 8.82
Communication and interaction capacity	0–36	17	33	21.07 ± 7.93
Willingness to improve health	0–16	6	15	10.01 ± 4.02
Willingness for financial support	0–8	2	8	4.88 ± 2.64

**Table 2:** Relevance (*r*) of depression, daily living ability, and health literacy scores.

	Basic daily living ability	Functional daily living ability	Health literacy	Depression
Basic daily living ability	1			
Functional daily living ability	0.610^a^	1		
Health literacy	0.398^a^	0.563^a^	1	
Depression	−0.200^a^	−0.298^a^	−0.374^a^	1

Note: ^*a*^*P* < 0.001.

**Table 3:** Tests of the intermediary variables of health literacy.

Step	Dependent variable	Independent variable	*B* value	SE	*β* value	*t* value	*R* ^2^	*F* value
Step 1	Daily activity ability	Depression	−0.408	0.027	−0.502	−10.821^a^	0.324	209.412
Step 2	Health literacy	Depression	−0.284	0.026	−0.446	−8.776^a^	0.184	96.078
Step 3	Daily activity ability	Depression	−0.276	0.033	−0.388^a^	−9.014^a^	0.521	232.416
		Health literacy	0.522	0.042	0.482^a^	12.084^a^		

Note: ^*a*^*P* < 0.001.

**Table 4:** Statistical table of model effect evaluation.

Number	Mental health improvement effect	Quality of life improvement effect	Customer satisfaction	Number	Mental health improvement effect	Quality of life improvement effect	Customer satisfaction	Number	Mental health improvement effect	Quality of life improvement effect	Customer satisfaction
1	84.1	79.7	90.9	21	84.1	82.5	94.4	41	89.7	85.4	93.9
2	88.2	87.3	92.1	22	82.7	99.9	87.6	42	83.3	94.0	89.4
3	78.7	79.9	96.1	23	81.3	84.7	93.5	43	84.9	99.7	96.9
4	78.3	79.7	86.0	24	89.1	85.1	93.1	44	91.3	94.7	93.5
5	88.0	86.2	89.3	25	82.6	84.3	93.9	45	87.4	82.1	89.0
6	82.1	88.1	91.5	26	90.1	97.8	92.2	46	86.1	81.7	88.2
7	80.1	95.9	87.7	27	78.8	96.6	96.7	47	85.4	97.5	94.0
8	86.7	90.9	93.1	28	89.3	80.4	86.3	48	86.8	84.2	86.0
9	89.7	95.7	96.6	29	78.6	85.3	94.1	49	84.7	86.7	94.5
10	88.5	86.3	89.2	30	83.2	79.9	96.1	50	84.3	80.9	96.4
11	89.7	95.7	92.9	31	88.5	99.9	92.5	51	88.6	99.1	96.9
12	85.7	90.3	89.3	32	90.6	93.1	94.6	52	89.7	99.2	92.1
13	90.9	80.9	87.3	33	80.2	97.2	95.4	53	91.0	88.4	92.4
14	89.3	89.0	96.9	34	84.8	98.6	93.5	54	80.2	96.6	85.2
15	82.9	88.0	86.6	35	86.2	92.7	95.8	55	78.4	82.7	88.0
16	86.7	95.7	88.6	36	82.9	84.7	92.2	56	82.2	92.1	93.0
17	88.8	84.0	92.5	37	81.7	93.6	93.8	57	84.3	99.8	89.1
18	86.6	86.7	88.4	38	80.5	90.7	93.9	58	91.3	92.0	92.3
19	88.9	80.4	90.8	39	91.8	87.2	94.8	59	80.2	88.8	91.6
20	90.4	87.8	85.1	40	84.3	95.3	86.7	60	90.2	85.7	89.8

## Data Availability

There is no analytical permission from the data provider because of trade confidentiality.
